# Real-world experience with tirbanibulin 1% ointment for the treatment of nonmelanoma skin cancer following cryotherapy: A pilot study

**DOI:** 10.1016/j.jdin.2024.11.005

**Published:** 2024-11-29

**Authors:** Angela Moore, Kara Hurley, Stephen Moore, Luke Moore

**Affiliations:** a Arlington Center for Dermatology, Arlington, Texas; b Arlington Research Center, Arlington, Texas; c Department of Dermatology, Baylor University Medical Center, Dallas, Texas; d Texas Christian University School of Medicine, Fort Worth, Texas; e University of North Texas Health Science Center Texas College of Osteopathic Medicine, Fort Worth, Texas; f Department of Dermatology, Texas Tech University Health Science Center, Lubbock, Texas

**Keywords:** basal cell carcinoma, clinical research, drug response, general dermatology, medical dermatology, nonmelanoma skin cancer, oncology, squamous cell carcinoma, tirbanibulin, topical

*To the Editor:* Tirbanibulin 1% ointment is a synthetic antiproliferative agent approved by the Food and Drug Administration for actinic keratosis (AK) treatment. Tirbanibulin exhibits a dual mechanism of action including microtubule proliferation inhibition as well as Src kinase signaling.^1^ Tirbanibulin 1% ointment has clinically eradicated periungual squamous cell carcinoma (SCC) and basal cell carcinoma (BCC) of the ear.^2^^,^^3^ In another case series, topical tirbanibulin has clinically and histologically resolved 6 out of 7 biopsy-proven SCC or SCC in situ (SCCIS).^4^ Now, we report real-world experience in a retrospective case series pilot study of 30 biopsy-proven SCC, SCCIS, or BCC treated off-label with tirbanibulin after 32 months of follow-up.

Patients underwent a varying number of treatment courses depending on clinical response. Each treatment course, or round, consisted of 5 consecutive days of once-nightly application followed by no application for 2 weeks. Each round was precipitated by enough liquid nitrogen for a light frost without producing a blister to facilitate deeper delivery of tirbanibulin. Clinical resolution was determined by clinically visible skin or pathology only if clinical resolution was equivocal. Local skin reactions (LSRs) were evaluated objectively by the dermatologist based on photographs by the patient before nightly applications combined with subjective reports by the patient. LSRs were graded as none, mild, moderate, or severe by the dermatologist.

Thirty non-melanoma skin cancer (NMSC) lesions were reviewed, consisting of 9 SCC, 17 SCCIS, 3 nodular BCC, and 1 morpheaform BCC. The sizes ranged from 5 to 14 mm (M = 9.24, SD = 2.86). Patient demographics and previous failed treatments are listed in Table I. The number of rounds required ranged from 1 to 7 (M = 3.2, SD = 1.85). Twenty-four of thirty (80%) NMSC resolved with tirbanibulin (95% CI [65%, 94%]) (Fig 1). No recurrence has been observed at 32 months.

**Table I:** Demographics and Clinical Data

Case #	Age/sex	Fitz	Race	Ethnicity	Biopsy-proven diagnosis	Location	Size	Failed IMQ	Failed 5-FU	Failed calcipotriene	Failed PDT	Failed LN2	# of tirbanibulin rounds	Clinical resolution	LSR	Recurrence after 32 months
1	82M	I	White	Non-Hispanic	SCC	Ear	9 mm	Yes	No	No	No	Yes	1	Yes	None	No
2	63F	II	White	Non-Hispanic	SCCIS	Cheek	8 mm	No	No	No	Yes	Yes	2	Yes	Moderate	No
3	76F	IV	White	Non-Hispanic	SCCIS	Cheek	1.2 cm	Yes	No	No	No	Yes	5	Yes	Mild	No
4	78M	III	White	Non-Hispanic	mBCC	Nose	9 mm	Yes	No	No	No	Yes	4	No	None	No
5	72F	II	White	Non-Hispanic	SCCIS	Lip	5 mm	Yes	No	No	No	Yes	6	Yes	Mild	No
6	72M	III	White	Non-Hispanic	SCCIS	R ear	7 mm	No	No	No	Yes	Yes	1	Yes	Mild	No
7	72M	III	White	Non-Hispanic	nBCC	L ear	9 mm	No	No	No	Yes	Yes	1	Yes	None	No
8	63M	II	White	Non-Hispanic	SCCIS	Scalp	1.2 cm	Yes	No	No	Yes	Yes	5	Yes	None	No
9	66F	II	White	Non-Hispanic	nBCC	Nose	9 mm	Yes	No	No	No	Yes	4	Yes	Mild	No
10	59M	II	White	Non-Hispanic	SCCIS	Temple	1.2 cm	No	No	No	No	Yes	2	Yes	None	No
11	52F	IV	White	Hispanic	SCCIS	Nose	8 mm	Yes	No	No	No	Yes	2	Yes	None	No
12	60F	II	White	Non-Hispanic	SCC	Chest	1.1 cm	No	Yes	Yes	No	Yes	1	Yes	Mild	No
13	60F	II	White	Non-Hispanic	SCCIS	Cheek	9 mm	No	No	No	No	Yes	6	Yes	Moderate	No
14	74F	III	White	Non-Hispanic	SCC	Hand	1.2 cm	Yes	No	No	No	Yes	1	Yes	Moderate	No
15	60M	III	White	Non-Hispanic	SCCIS	Nose	5 mm	No	No	No	No	Yes	2	Yes	Mild	No
16	79M	II	White	Non-Hispanic	SCCIS	Temple	1.2 cm	Yes	No	No	No	Yes	3	Yes	None	No
17	60F	III	White	Non-Hispanic	SCCIS	Nose	1.2 cm	No	No	No	No	Yes	3	Yes	Mild	No
18	52M	III	White	Non-Hispanic	SCC	Arm	9 mm	No	Yes	No	No	Yes	7	Yes	Mild	No
19	78F	II	White	Non-Hispanic	SCCIS	Scalp	1.3 cm	No	Yes	No	No	Yes	1	Yes	None	No
20	64M	II	White	Non-Hispanic	SCC	Cheek	1.2 cm	Yes	No	No	No	Yes	3	Yes	Mild	No
21	55F	II	White	Non-Hispanic	nBCC	Chest	9 mm	Yes	No	No	No	Yes	5	Improved	None	No
22	72F	II	White	Non-Hispanic	SCCIS	Nose	1.1 cm	No	No	No	No	Yes	6	Improved	Moderate	No
23	72F	II	White	Non-Hispanic	SCCIS	Chest	9 mm	Yes	No	No	No	Yes	5	Yes	Mild	No
24	21F	II	White	Non-Hispanic	SCCIS	Hand	9 mm	No	No	No	No	Yes	5	Improved	Mild	No
25	55F	V	Black	Non-Hispanic	SCC	Ear	8 mm	Yes	No	No	No	No	1	Yes	Severe	No
26	74M	II	White	Non-Hispanic	SCC	Temple	1.4 cm	Yes	No	No	Yes	Yes	3	No	None	No
27	63F	III	White	Non-Hispanic	SCC	Chest	1.4 cm	No	No	No	Yes	Yes	4	Yes	Mild	No
28	59F	III	White	Non-Hispanic	SCC	Cheek	6 mm	No	No	No	Yes	No	1	Yes	Mild	No
29	69F	II	White	Non-Hispanic	SCCIS	Forehead	7 mm	No	No	No	Yes	No	2	Yes	Mild	No
30	74M	II	White	Non-Hispanic	SCCIS	Lip	6 mm	Yes	No	No	Yes	Yes	3	No	None	No

*5-FU*, Fluorouracil; *Fitz*, fitzpatrick skin type; *IMQ*, imiquimod; *LN2*, liquid nitrogen cryotherapy; *LSRs*, local skin reactions; *mBCC*, morpheaform basal cell carcinoma; *nBCC*, nodular basal cell carcinoma; *PDT*, photodynamic therapy; *SCC*, squamous cell carcinoma; *SCCIS*, squamous cell carcinoma in situ.

**Fig 1:**
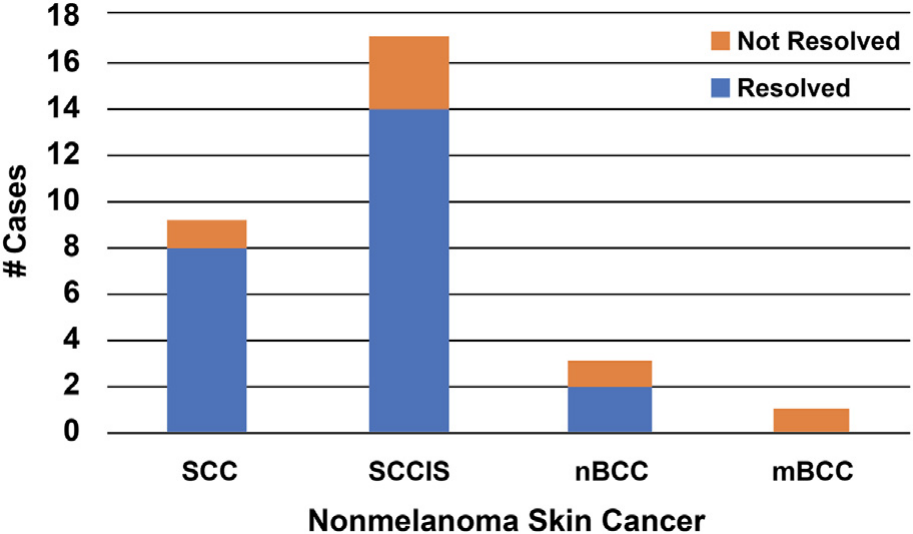
Nonmelanoma skin cancer response to tirbanibulin by subtype. The demographics and details of the represented patients can be found in Table I. *mBCC*, Morpheaform basal cell carcinoma; *nBCC*, nodular basal cell carcinoma; *SCC*, squamous cell carcinoma; *SCCIS*, squamous cell carcinoma in situ.

In any NMSC with equivocal clinical resolution, complete excision was performed. Confirmatory excision of 1 SCC demonstrated residual SCC but excision of 3 SCC demonstrated no residual SCC histologically. Two patients with clinically resolved SCCIS opted for confirmatory excision; both cases showed no residual SCCIS histologically. The morpheaform BCC was excised through Mohs surgery. LSR was graded as mild or none in 83% (Table I). Greater LSR resulted from thicker application of tirbanibulin and correlated with quicker onset of efficacy. However, LSR was not mandatory for clinical efficacy.

Some patients with NMSC are poor surgical candidates, refuse surgical intervention, or request nonsurgical treatments prior to excision. Topical agents such as 5-fluorouracil and imiquimod have limited effectiveness (especially in SCC) and often severe LSRs, which then decreases compliance. One review of topical therapies for AK treatment noted better tolerability and infrequent severe LSRs with tirbanibulin.^5^ In our case series, patients that opted for off-label treatment with tirbanibulin 1% ointment usually had extensive NMSC history and requested topical therapy prior to excision. Given our findings in this pilot study on a retrospective case series of 30 biopsy-proven SCC, SCCIS, or BCC treated off-label with tirbanibulin after 32 months of follow-up, larger prospective, double-blinded studies of tirbanibulin for NMSC treatment, especially SCC or SCCIS, are warranted.

## Conflicts of interest

Dr Angela Yen Moore has received honoraria and/or research funds from Almirall, LLC. Drs Hurley, Stephen Moore, and Luke Moore have no conflicts of interest to declare.
